# A Mortality Prediction Rule for Hematology Patients with Invasive Aspergillosis Based on Serum Galactomannan Kinetics

**DOI:** 10.3390/jcm9020610

**Published:** 2020-02-24

**Authors:** Toine Mercier, Joachim Wera, Louis Y. A. Chai, Katrien Lagrou, Johan Maertens

**Affiliations:** 1 KU Leuven, Department of Microbiology, Immunology and Transplantation, 3000 Leuven, Belgium; Katrien.lagrou@uzleuven.be (K.L.); johan.maertens@uzleuven.be (J.M.); 2 Department of Hematology, University Hospitals Leuven, 3000 Leuven, Belgium; joachim.wera@uzleuven.be; 3 Division of Infectious Diseases, University Medicine Cluster, National University Health System, Singapore and Yong Loo Lin School of Medicine, National University of Singapore, Singapore 119074, Singapore; chailouis@hotmail.com; 4 Department of Laboratory Medicine and National Reference Center for Mycosis, University Hospitals Leuven, 3000 Leuven, Belgium

**Keywords:** invasive aspergillosis, galactomannan, kinetics, prognosis, outcome, mortality

## Abstract

In invasive aspergillosis (IA), an early and adequate assessment of the response to the initial antifungal therapy remains problematic. We retrospectively analyzed 206 hematology patients with proven or probable IA, and collected serial serum galactomannan (sGM) values and survival status through week 6 and week 12. We created a model for survival at week 6 based on the sGM taken at baseline and on early sGM kinetics. This resulted in a rule predicting that patients with a baseline sGM index >1.4, who failed to lower that index to <0.5 after one week, had a mortality rate of 48.1% at week 6. Conversely, patients presenting with a baseline sGM index ≤1.4 that obtained a negative sGM (<0.5) after one week, had a mortality that was almost five times lower at only 10.1% by week 6. These findings were confirmed in an external cohort from an independent prospective study. In conclusion, sGM kinetics correlate well with treatment outcomes in hematology patients with IA. We present a rule which is easy to use at the bedside and has good accuracy in predicting week 6 survival.

## 1. Introduction

Invasive aspergillosis (IA) is a life-threatening disease in hematology patients, especially in those with deep and prolonged neutropenia following myelotoxic chemotherapy and in those receiving immunosuppressive therapy for treatment of graft-versus-host disease after allogeneic hematopoietic cell transplantation [1]. Early diagnosis remains difficult, especially when relying on conventional tools such as microscopic examination and fungal culture, resulting in delayed treatment and significant morbidity and mortality [2,3]. 

Galactomannan (GM) is a cell wall polysaccharide of Aspergillus species (and some other fungi), which is released by growing hyphae during tissue invasion. Detection of circulating GM by a commercial sandwich-enzyme immunosorbent assay has become an important tool for the diagnosis of IA. This is underscored by the inclusion of GM as a microbiological criterion in the European Organization for Research and Treatment of Cancer/Mycoses Study Group (EORTC-MSG) consensus definitions for invasive fungal diseases [4]. The diagnostic performance and clinical utility of GM measurements has been studied in different clinical settings and has been the subject of several meta-analyses [5,6,7,8]. Nowadays, hematologists use this assay primarily as a diagnostic test (e.g., on broncho-alveolar lavage (BAL) fluid in patients with unexplained pulmonary lesions) or, in neutropenic patients, as a screening tool on serum samples. 

Despite some recent advances in diagnosing the infection, the early assessment of response to antifungal therapy remains problematic. Historically, a composite endpoint of clinical, radiological, and microbiological outcomes has been used [9]. However, clinical assessments are often subjective and based on non-specific signs and symptoms. Even the presumed objective assessments have significant limitations: on follow-up imaging studies, the initial fungal lesions often increase in size during the first (two) weeks; although this may indicate a poor treatment outcome, it more often reflects the expected course of the infection or it may be due to immune reconstitution following neutrophil recovery or tapering of the immunosuppression [10]. In addition, serial microbiologic assessments are impractical if an invasive procedure such as repeated bronchoscopic lavage and/or biopsy is required. Therefore, a biomarker that can predict clinical outcome, particularly early after starting antifungal therapy (e.g., after 1 week), would be a valuable tool for clinicians to guide their antifungal treatment decisions.

Galactomannan could be such a biomarker. Indeed, animal studies have consistently demonstrated a strong correlation between baseline serum galactomannan (sGM) levels and sGM kinetics and biological outcome variables, such as quantitative cultures (fungal burden), organism-mediated pulmonary injury (measured by lung weight, infarct scores, and CT scan scores), survival, and treatment response [11,12,13]. Therefore, a decrease in sGM correlates with a decreased fungal burden. However, demonstrating a similar strong correlation in patients appears more challenging. Besides, clearance of sGM occurs through several mechanisms, including elimination via neutrophils, hepatic uptake through macrophages (Kuppfer cells), and renal clearance [14]. Furthermore, the class-specific mechanism of action of the different antifungal drugs could also alter the release of GM (e.g., echinocandins induce cell wall lysis), whereas all mold active agents interfere with the test performance [8,15]. This demonstrates a complex interplay between sGM levels, fungal burden, antifungal therapy, and several host factors such as neutropenia and comorbidities. It is therefore essential to study the in vivo kinetics of sGM and its significance after initiation of antifungal therapy in a real-life patient population. 

In this study, we assessed the correlation between sGM levels during the first week of antifungal treatment and clinical outcomes (survival and response) through day 42 (week 6) in patients with documented IA. We further created an easy-to-use prediction rule for week 6 mortality based on sGM values determined at baseline and after one week of anti-Aspergillus therapy.

## 2. Experimental Section

### 2.1. Study Design

This retrospective, non-interventional study was conducted at a single academic centre in Belgium (the Belgian National Reference Center for Mycosis, University Hospitals Leuven, Leuven, Belgium). The study was approved by the institutional Ethics Committee. 

#### 2.1.1. Data Collection

We reviewed the electronic medical files of (a) all adult in-hospital patients that had at least one GM test performed on serum or BAL fluid between 1 January 2011 and 31 December 2016 and (b) all patients prospectively included in a hospital registry of hematology patients with proven and probable IA between 1 January 2001 and 31 December 2010. Only patients with at least two consecutive serum samples with an optical density index (ODI) ≥ 0.5 on separate days, and/or GM ODI ≥ 0.5 on BAL fluid, were included. Patients were considered evaluable for further analysis if they had (1) proven or probable IA, (2) received mold-active antifungal therapy, and (3) outcome data were available. When a patient had multiple episodes of IA, only the first episode was considered for analysis.

For all evaluable patients, we recorded type of antifungal therapy, use of mold-active prophylaxis, weight, and height. Baseline laboratory tests (serum liver enzymes, creatinine, absolute neutrophil count, monocyte count, and platelet count) were recorded at baseline ±3 days. 

An existing dataset from a large international study on voriconazole–anidulafungin combination therapy in hematology patients (NCT00531479) served as a validation cohort [16]. We collected age, gender, BMI, underlying disease, final EORTC/MSG classification, and survival at week 6, as well as baseline and week 1 serum galactomannan.

#### 2.1.2. Definitions

Patients were classified per the 2008 EORTC-MSG consensus definitions [4]. Because a positive serum or BAL GM was used to identify patients for this study, no cases had possible IA. Baseline (day 0) was defined as the first day of systemic mold-active therapy. 

#### 2.1.3. Outcome Measurements

Patient outcomes were assessed at week 6 and 12 after the start of anti-Aspergillus therapy. All-cause mortality at week 6 was the primary outcome, as IA-attributable mortality wanes thereafter [17]. 

#### 2.1.4. Galactomannan Testing

GM testing was done thrice weekly by the National Reference Center for Mycosis using the Platelia™ Aspergillus enzyme immunoassay (Bio-Rad Laboratories, Marnes-la-Coquette, France). Results are reported with a 0.1 precision (i.e., positive tests have a value from 0.5 and higher, negative tests a value of 0.4 or lower). In hematology patients at high risk of IA (patients receiving intensive chemotherapy for acute leukemia or myelodysplastic syndrome, patients receiving immunosuppressive therapy for aplastic anemia, and allogeneic hematopoietic cell transplant recipients), daily sampling of sGM is a key element of our care pathway during episodes of prolonged and profound neutropenia and during graft-versus-host disease. Two consecutive positive assays (sGM ≥ 0.5) is one of the triggers for ordering a chest CT, followed by bronchoscopy and lavage in case of suggestive features of pulmonary infection. Since 2010, testing for BAL GM has become standard of care for hematology patients, transplant recipients, and critically ill patients presenting with unexplained pulmonary lesions. 

### 2.2. Statistical Mmethods

The goal of this study was to create a clinical decision rule to predict the outcome based on baseline and week 1 sGM. This implies the need for a specific cut-off for either the absolute values at both points in time, or the relative or absolute change after one week. To allow the use of all cases in our multivariate analysis, we imputed missing data using multiple imputation by chained equations, so as to avoid complete-case analysis bias. This form of imputation uses regression modeling to impute missing variables based on the remaining variables, instead of just substituting, for example, the mean or median [18]. Next, we checked for any interaction between baseline and week 1 by both variables as continuous using three splines in a logistic regression with week 6 survival as outcome. If a significant interaction was found between two parameters, the cut-offs for these parameters should be determined simultaneously instead of using a stepwise approach. The cut-offs with the highest discriminatory power as determined by the Youden index were selected for further analysis. The Youden index is a single-number parameter for the overall performance of a test based on the sensitivity and specificity (Youden index = sensitivity + specificity − 1) [19]. This means that a perfect test would have a Youden index of 1, a purely random test would have an index of 0, and a test that performs worse than random chance would have a negative index. 

We used multivariate logistic regression to assess the impact of this newly created rule after controlling for age, gender, EORTC/MSG classification, body mass index, class of the initial antifungal agent (azole, echinocandin or polyene), EORTC/MSG classification (proven versus probable), use of mold-active prophylaxis, bilirubin level, creatinine level, prolonged use of corticosteroids (prednisone-equivalent ), pre-existing cardiac disease, diabetes mellitus requiring treatment, and neutropenic state (absolute neutrophil count <500/µL). All statistics were calculated using R version 3.6.1 (R Foundation for Statistical Computing, Vienna, Austria)**.**

## 3. Results

### 3.1. Patient Disposition and Baseline Characteristics

**Figure 1:**
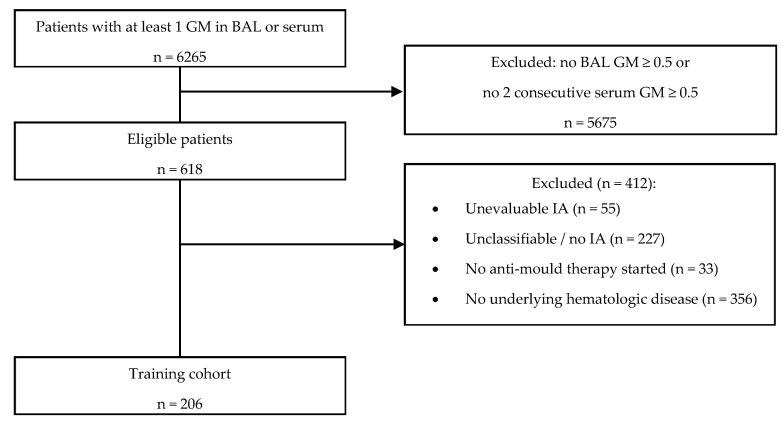
Flowchart of subject selection. Reasons for exclusion are not mutually exclusive. BAL = bronchoalveolar lavage. GM = galactomannan. IA = Invasive aspergillosis.

**Figure 2:**
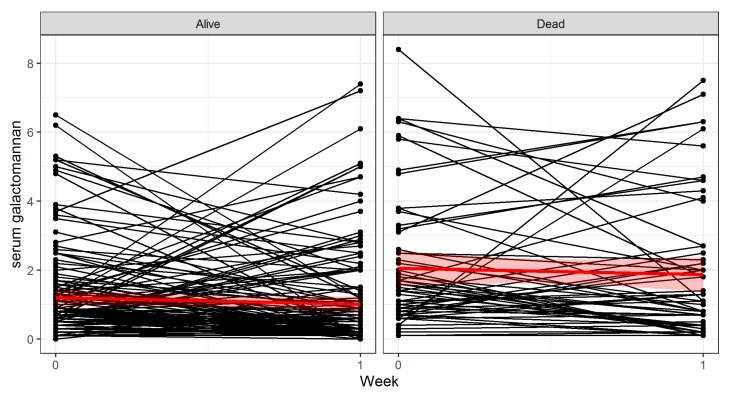
Evolution of serum galactomannan from baseline to week 1. The red line shows the averaged evolution, with the standard error marked in light red.

The patient disposition is shown in Table 1, and the flow of study subjects is depicted in Figure 1. A total of 206 patients were evaluable for the training cohort. The overall 6 week mortality in this cohort was 28.6%. The evolution of each patient is shown in Figure 2.

### 3.2. Model Creation

We chose to train our model using week 6 mortality as outcome as EORTC/MSG-defined global responses do not always correlate with survival [20,21]. This discordance can be caused by slow resolution of radiographic lesions or by the appearance of new lesions not due to IA. Indeed, visual inspection of sGM ODI trends, stratified by survival and by response at week 6 and week 12 (data not shown), showed the largest separation in trends when stratifying by survival at week 6. Week 12 survival is frequently influenced by the status of the underlying disease or other infections, as previously demonstrated [17]. As such, week 6 survival appeared to be the best outcome measure.

**Figure 3:**
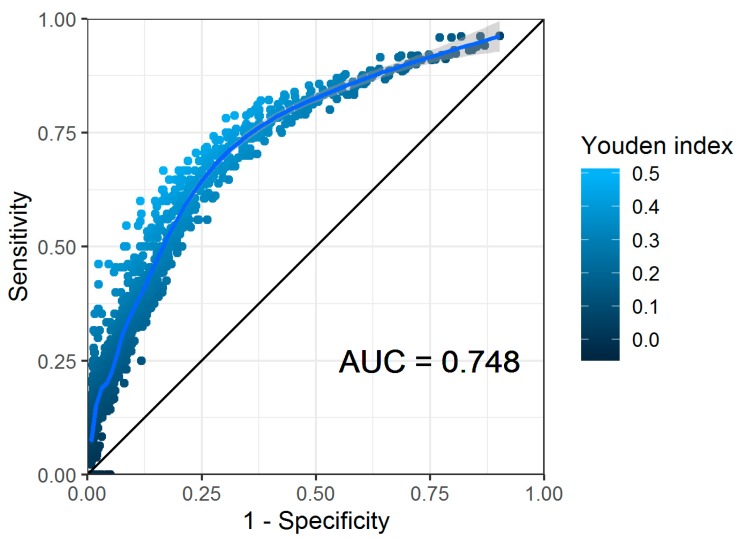
Receiver operating characteristic (ROC) plot of all baseline and week 1 serum galactomannan values. Each dot represents a unique combination of cut-off points for each value. The area under the curve (AUC) is provided for the smoothed generalized additive model regression line, drawn in light blue.

We found a significant interaction (*p* = 0.007) between the absolute sGM values at baseline and at week 1 (but not for relative or absolute decline), and thus determined the cut-offs for both parameters simultaneously (Figure 3). 

In this analysis, the highest Youden index of 0.48 was obtained using a baseline cut-off of 1.4 and week 1 cut-off of 0.4, resulting in a sensitivity of 0.78 (95% confidence interval (CI) 0.60–0.91) and a specificity of 0.70 (95% CI 0.59–0.79) for predicting mortality at week 6. For the other parameters, the optimal cut-off was determined based on the Youden index, independent of the cut-offs of the other parameters taken into consideration (Table 2).

**Figure 4:**
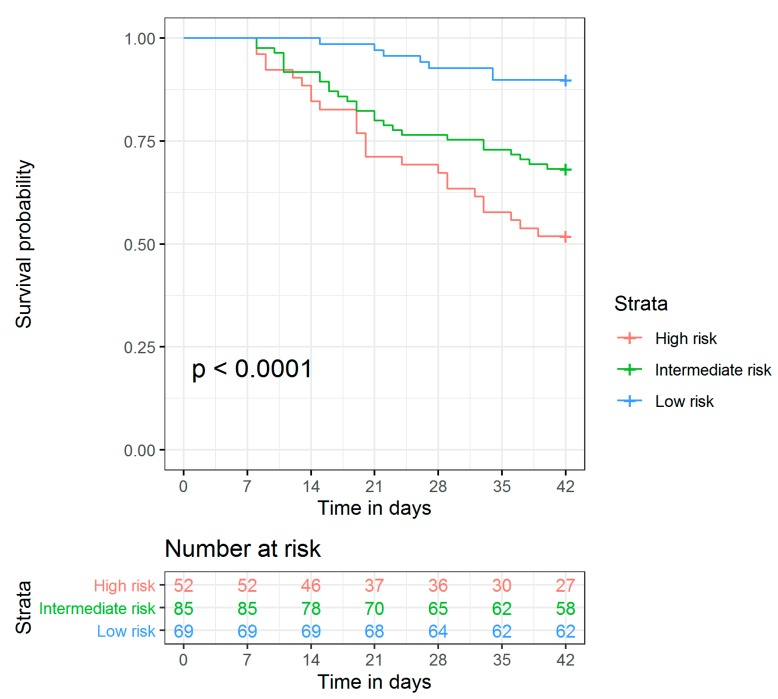
Survival curves for patients with high, intermediate or low risk of mortality, based on serum galactomannan values at baseline and week 1.

As the combined use of absolute sGM values at baseline and week 1 had the best performance by far, we selected this kinetic rule for further evaluation. Based on this rule, we would expect a patient with a high sGM at baseline (ODI > 1.4) that remains positive by week 1 (ODI > 0.4) to have a high mortality. Conversely, a patient with a low sGM at baseline (ODI ≤ 1.4) that lowers to or remains at a negative level (ODI ≤ 0.4) to have a low mortality. Patients not fitting in either of these groups (i.e., low baseline sGM but not negative after 1 week, or high baseline sGM but becoming negative after 1 week) are expected to have an intermediate mortality. Indeed, the 6 week cumulative mortality was 48.1% in the high risk group, 31.8% in the intermediate risk group, and 10.1% for the low risk group (Figure 4). In multivariate analysis, this classification was a statistically significant independent predictor for mortality (*p* = 0.003). The baseline characteristics, stratified by risk group allocation, are shown in Table 3.

The intermediate risk group consists of two distinct groups of patients: on the one hand patients that start out with a high sGM > 1.4 but become negative after one week, and on the other hand, patients that start out with a “low” sGM ≤ 1.4 but that fail to become negative after one week. In this risk group, the mortality in patients who had a baseline sGM ≤ 1.4 but that managed to achieve a negative sGM after one week was 29% (5/17), compared to 33% (22/67) in those who started out with an sGM ≤ 1.4 but did not achieve a negative sGM after one week (*p* = 1.000).

### 3.3. Model Validation

In this external validation cohort, the overall mortality at week 6 was 22.8% (versus 28.6% in the training cohort, *p* = 0.171). Based on the above prediction rule, 69 patients were classified as high risk with a 6 week mortality of 40.6%, 80 as intermediate risk (mortality 15.0%), and 136 as low risk (mortality 18.4%). There was no statistically significant difference between the mortality in the low risk and intermediate risk groups (*p* = 0.579). However, there was a large and statistically significant difference between the 6 week mortality in the high risk group versus the intermediate risk group (*p* < 0.001) and between the high risk group and the low risk group (*p* = 0.001). When combining the intermediate and low risk groups, the difference remains significant (6 week mortality 17.1% versus 40.6%, *p* < 0.001).

## 4. Discussion

We created a simple prediction rule for week 6 mortality in hematology patients with IA based on the baseline and week 1 sGM levels. It predicts that patients that have a sGM ODI > 1.4 and that fail to attain a negative sGM (ODI < 0.5) after one week will have a high mortality. Conversely, patients that have a low sGM (ODI ≤ 1.4) at diagnosis and are negative (ODI < 0.5) after one week will have a low mortality. This rule is easy to use at the bedside, without the need for multiple covariates or computer models, while still predicting a doubling in mortality after one week after treatment based on a single blood test. Visual analysis of Figure 2 shows where this model finds its basis: patients that survive by week 6 generally start out with a low sGM index at baseline and remain low by week 1. Patients that have died by week 6 started out with a high baseline sGM index on average and failed to decrease this index by week 1. This model appears to be robust and not the result of overfitting, as evidenced by the validation in an independent patient population, as well as from the multivariate regression. This is further exemplified by Table 3, which shows no difference in baseline characteristics between the three groups, with the exception of EORTC/MSG classification. There were statistically less cases of proven IA in the low risk group, likely due to survivorship bias: the diagnosis of proven IA was often only made at autopsy. A second significant difference between the subgroups was in the number of patients that fulfills the 2019 revision of the EORTC/MSG criteria [22]. Indeed, this study was designed and performed before these criteria were published. A post hoc analysis revealed that a significant number, though not all patients would still be classified as having probable IA. This number was significantly higher in the high risk subgroup (100%) as by definition, patients need to have a baseline sGM > 1.4 to be in this subgroup, fulfilling the new mycological criteria. This was not obligatory in the low and intermediate risk groups. In the subgroup of only patients fulfilling the new criteria, the risk stratification remained significant: survival was 53% in the high risk group, 66% in the intermediate group, and 93% in the low risk group (*p* < 0.001, *n* = 169).

The robustness of our prediction rule is further supported by previous, independent studies that found similar cut-offs and correlations to the ones defined by our mathematical model [23]. The outcome of our prediction rule could therefore help clinicians in deciding if treatment is successful early on, after only one week of treatment. If treatment failure is anticipated, possible causes should be explored, including drug failure (e.g., resistance to the antifungal agent or inadequate exposure) and/or clinical failure due to ongoing defects in the host immunity.

An interesting effect can be seen in the intermediate risk group. Instinctively, an increasing sGM after 1 week despite therapy would suggest therapy failure and might appear worse than a baseline sGM ≤ 1.4 that fails to become negative after one week. However, it appears that a very high initial serum GM remains an important predictor of outcome, even if this then drops quickly, with an effect of roughly the same size as a rising serum GM after initial low GM. 

Previous studies have shown a correlation between sGM ODI at baseline, kinetics, and outcome [23]. However, we could identify only two studies that proposed a prediction rule based on sGM ODI kinetics at 1 week [24,25]. Kovanda et al. found that an increase of sGM ODI by day 7 of >0.25 was associated with a 10-fold increase in risk of death, when compared to smaller increases, stable results, or declining sGM ODI [25]. However, as this study only included patients that were sGM positive at baseline, this rule is not applicable in patients that have a negative sGM at baseline, but become positive by week 1 (which would be classified as intermediate risk in our model). Chai et al. found that in patients who were sGM positive at baseline, a decline in sGM index of >35% by week 1 was correlated with good outcome [24]. However, this study only reported a correlation with week 12 clinical outcome, whereas we applied it to week 6 survival. Neither study used an independent cohort to validate their findings. We therefore applied these rules to our training cohort, but could not confirm their results: the 6 week mortality was not significantly different in the high risk group from the low risk group in the training cohort (*p* = 0.200 for the rule by Chai et al., and *p* = 0.170 for the rule by Kovanda et al.) or in the external validation cohort (*p* = 0.570 for the rule by Chai et al., and *p* = 0.053 for the rule by Kovanda et al.). One possible explanation for this discrepancy with the results by Kovanda et al. is that in that study sGM values were not tested per protocol at 1 week. This resulted in a large number of missing values, which were then interpolated from baseline and week 2 levels. However, it is not known what the actual kinetics are in this time interval, which could lead to overinterpretation of interpolated values.

Our prediction rule was mainly evaluated in neutropenic hematology patients. Therefore, generalization of our results to other at-risk populations, including solid organ transplant recipients, should be done cautiously, as there are differences in host response to the infecting mold, and due to differences in clearance of sGM by the immune system, which ultimately results in lower levels of sGM overall [5,8]. Creating a general model of sGM kinetics remains difficult due to high variability in metabolism given the different elimination pathways [26]. Furthermore, creating a perfect prediction rule based on a single biomarker remains a challenge, as other factors, such as the underlying disease, influence the outcome at week 6 in this highly comorbid population. As such, our model is not meant to eliminate all other factors that guide clinical decision making or replace clinical expertise from an experienced physician. In fact, we aim to offer the clinician an additional, robust and simple to use tool to be used in conjunction with all other data available. For example, imaging can be deceiving in the early course of therapy [10]. As such, our risk stratification could help in cases where radiology is stable or worsening, yet the patient is improving clinically.

The generalizability of our study will also depend on the exact anti-fungal approach used, as our study was based on data from a population in which sGM screening was used in patients at the highest risk for IA, without antifungal prophylaxis. In case prophylaxis is used, we expect baseline GM to be lower in general. This would mean that if a patient attains sGM > 1.4 despite prophylaxis, mortality will likely be significantly higher. In such populations, the cut-offs would likely have to be lower. In populations where screening is not used, but rather a symptom-based approach, we would expect higher initial serum GM as screening for GM can detect IA before the onset of symptoms [27]. However, to the best of our knowledge, there are no interventional trials comparing treatment outcomes in patients undergoing screening compared to patients managed using a symptom-driven approach.

A major advantage of our study is the fact that we had access to more than 9500 daily sGM values after initiation of therapy, did not rely on modeled or interpolated values, and included only probable and proven cases, in contrast to previous studies. Another strength of our model is the use of discrete cut-off points (as opposed to a continuous risk estimate), which eliminates any possible interference of inter-testing variability which can be seen at higher sGM values due to the influence of the nonlinear range inherent to photometry at higher densities [23]. Furthermore, our prediction rule is not limited to patients who have a positive sGM at baseline, as our model was trained using data from patients who had positive GM in serum or BAL. This improves the generalizability of our model and allows its use within the complete hematologic population with IA. This is also supported by previous studies showing improved survival in patients with negative sGM at baseline [28,29,30,31].

## 5. Conclusions

In this study, we demonstrated distinct sGM kinetics between surviving and non-surviving hematology patients with invasive aspergillosis. We present a rule for predicting week 6 survival, based on these kinetics, that is easy to use at the bedside. This rule may help clinicians to steward and monitor antifungal therapies and to predict treatment outcomes, both in clinical practice and in clinical studies. 

## Figures and Tables

**Table 1:** Patient characteristics of the training and validation cohort.

	Training Cohort	Validation Cohort	*p*-Value
*n*	206	285	
Age, years (median [IQR])	58.66 [49.30, 67.22]	55.00 [42.00, 63.00]	<0.001
BMI, kg/m^2^ (median [IQR])	23.61 [20.80, 27.12]	23.30 [20.60, 26.35]	0.699
Male gender (%)	129 (62.6)	161 (56.5)	0.204
Proven IA (%)	32 (15.5)	13 (4.6)	<0.001
Bilirubin, mg/dL (median [IQR])	0.66 [0.47, 0.97]		
Creatinine, mg/dL (median [IQR])	0.70 [0.54, 1.03]		
Neutrophils/µL (median [IQR])	0 [0, 845]		
Monocytes/µL (median [IQR])	0 [0, 100]		
Platelets/µL (median [IQR])	39,000 [20,250, 71,500]		
Active GvHD (%)	28 (13.7)		
High dose corticosteroids * (%)	46 (22.4)		
Initial antifungal therapy (%)			
Azole	113 (54.9)		
Echinocandin	48 (23.3)		
Polyene	32 (15.5)		
Combination	11 (5.3)		
Other	2 (1.0)		

* Defined as prednisone-equivalent dose of 0.3 mg/kg/day for 21 days. IQR = Interquartile range. BMI = Body mass index. IA = Invasive aspergillosis. GvHD = Graft-versus-host disease.

**Table 2:** Test characteristics of standalone and combined cut-offs for serum galactomannan for predicting mortality at week 6.

Test	Baseline Cut-off	Week 1 Cut-off	Sensitivity (95% CI)	Specificity (95% CI)	Youden Index
Baseline sGM	1.0	-	0.64 (0.51–0.76)	0.65 (0.56–0.72)	0.29
Relative sGM decline by W1	-	−1.5	0.07 (0.02–0.16)	0.88 (0.81–0.93)	−0.05
Absolute sGM decline by W1	-	0.8	0.78 (0.71–0.85)	0.32 (0.21–0.46)	0.10
Baseline + relative sGM decline by W1	1.0	−1.5	0.00 (0.00–0.2.)	0.94 (0.87–0.98)	−0.06
Baseline + absolut sGM decline by W1	1.0	0.8	0.00 (0.00–0.18)	1.00 (0.83–1.00)	0.00
Baseline and absolute sGM value at baseline and W1	1.4	0.4	0.78 (0.60–0.91)	0.70 (0.59–0.79)	0.48

sGM = serum galactomannan. W1 = week 1. CI = confidence interval.

**Table 3:** Baseline characteristics, stratified by risk group.

	High Risk	Intermediate Risk	Low Risk	*p*
*n*	54	84	68	
Proven IA (%)	17 (31.5)	12 (14.3)	3 (4.4)	<0.001
Initial Therapy (%)				0.209
Azole	29 (53.7)	39 (46.4)	45 (66.2)	
Echinocandin	14 (25.9)	20 (23.8)	14 (20.6)	
Combination	4 (7.4)	6 (7.1)	1 (1.5)	
Polyene	7 (13.0)	17 (20.2)	8 (11.8)	
Other	0 (0.0)	2 (2.4)	0 (0.0)	
Prophylaxis (%)	3 (5.6)	3 (3.6)	3 (4.4)	0.856
Male Gender (%)	32 (59.3)	56 (66.7)	41 (60.3)	0.605
Bilirubin, mg/dL (median [IQR])	0.78 [0.52, 1.19]	0.75 [0.50, 1.11]	0.64 [0.45, 0.90]	0.077
Creatinine, mg/dL (median [IQR])	0.74 [0.52, 1.11]	0.66 [0.52, 1.01]	0.68 [0.56, 0.91]	0.845
New mycological criteria * (%)	54 (100.0)	76 (96.2)	40 (74.1)	<0.001
High dose corticosteroids (%)	15 (27.8)	18 (21.4)	13 (19.1)	0.505

* Mycological criteria in accordance with the 2019 revision of the EORTC/MSG definitions of invasive fungal infections. IA = invasive aspergillosis, IQR = interquartile range.
